# Supplementary material for: Mortality in Via Ferrata Emergencies in Austria from 2008 to 2018
Source: Int J Environ Res Public Health. 2019 Dec 22;17(1):103. doi: 10.3390/ijerph17010103 (PMC6981439; doi:10.3390/ijerph17010103)
Supplement: Supplementary file 1 [file ijerph-17-00103-s001.pdf]

Figure S1. CONSORT flowchart of data acquisition in via ferrata accident

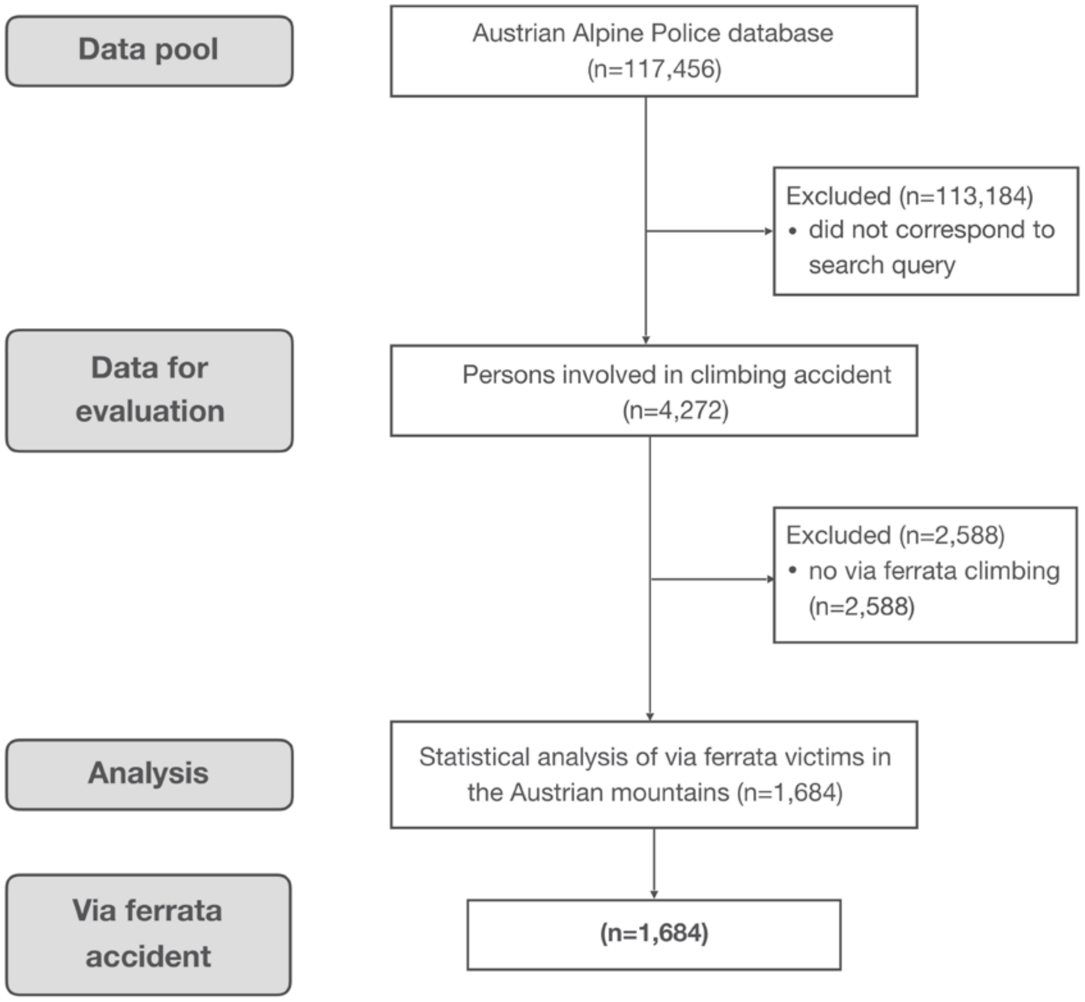

Figure S2. Case Report Form

Case ID: climbing accident in mountainous environment; Ver 1.0 1.10.2018

Case report Form climbing accident in mountainous environment

Inclusion Criteria:

- Person involved in climbing accident (female/male, 0-120 years of age)

Exclusion Criteria:

- hiking in steep environment

1.	**Person responsible for registration**		
**I**	**Case**		
2.	Patient identifier	ID number	
3.	Sex	☐ male<br>☐ female	
4.	Year of birth		
5.	Nationality		
**II**	**Accident and mission**		
6.	Date and time of accident	date / /	time /
7.	Rescue alarm	date / /	time /
8.	Primary cause of accident	☐ avalanche/ice fall<br>☐ cardiovascular disease<br>☐ crash into structure or terrain<br>☐ fall<br>☐ material failure<br>☐ neurological disorder	☐ thunderstorm<br>☐ security failure<br>☐ rockfall<br>☐ other medical condition:<br>☐ unknown
9.	Activity	☐ artificial wall climbing<br>☐ bouldering<br>☐ ice climbing<br>☐ rock climbing	☐ via ferrata<br>☐ other:<br>☐ unknown
10.	Difficulty of Route/Track		
11.	Difficulty of Accident Site and further Specification of Accident Site		
12.	Altitude of accident site	☐ <1500m<br>☐ 1500-2500m	☐ >2500m<br>☐ unknown
13.	Direction of Movement	☐ lead climb<br>☐ second climb<br>☐ top rope<br>☐ abseiling	☐ ascent, not further known<br>☐ descent, not further known<br>☐ other:
14.	Protective Gear		
15.	Weather Conditions (sunny, rain,…)		
16.	Guided Tour	☐ yes	☐ no
17.	Cell Phone	☐ yes	☐ no
18.	Circumstances of Accident (short report)		
**III**	**Out-of hospital care**		
19.	Duration before Emergency Medical Service (EMS)/mountain rescue (MR) arrival	____ min	☐ unknown
20.	First treatment before EMS/MR arrival	☐ no care<br>☐ first aid	☐ other:<br>☐ unknown
21.	Initial assessment of injury severity by EMS/MR	☐ unhurt<br>☐ minor injuries	☐ major injuries<br>☐ dead
22.	Initial treatment by EMS/MR	☐ immobilization<br>☐ first aid<br>☐ analgesics<br>☐ basic life support (BLS)	☐ advanced life support (ALS)<br>☐ other:<br>☐ unknown

Version 1.0 Page 1

Case ID: climbing accident in mountainous environment; Ver 1.0 1.10.2018

**IV**	**Primary transport**		
23.	Arrival at scene	date /	
24.	Departure time from scene	time /	
25.	Arrival time at hospital	time /	
26.	Mission characteristics	☐ air rescue<br>☐ terrestrial rescue<br>☐ terrestrial rescue with air support	☐ other:<br>☐ unknown
27.	Treatment during transport	☐ observation<br>☐ fluids<br>☐ analgesics<br>☐ anesthesia	☐ BLS<br>☐ ALS<br>☐ other:<br>☐ unknown
28.	NACA Score		
**V**	**First Hospital**		
29.	Name of the Hospital		
30.	Further examinations	☐ computed tomography scan<br>☐ magnetic resonance imaging<br>☐ radiography	☐ ultrasound<br>☐ other:
31.	Traumatic injuries (diagnoses)		
32.	Location of injuries	☐ head<br>☐ face<br>☐ cervical spine<br>☐ thoracic spine<br>☐ lumbar spine<br>☐ chest<br>☐ abdomen	☐ pelvis<br>☐ shoulder<br>☐ arm<br>☐ hand<br>☐ leg<br>☐ foot<br>☐ other:
33.	Abbreviated Injury Severity Score (AIS)	__ (0-6) and region:<br>☐ unknown	
34.	Abbreviated Injury Severity Score (AIS)	__ (0-6) and region:<br>☐ unknown	
35.	Abbreviated Injury Severity Score (AIS)	__ (0-6) and region:<br>☐ unknown	
36.	Injury Severity Score (ISS)	__ (0-75)<br>☐ unknown	
37.	In hospital treatment	☐ no treatment needed<br>☐ out-patient treatment<br>☐ care in the ward<br>☐ immobilization<br>☐ radiological intervention	☐ endoscopic intervention<br>☐ surgery:<br>☐ intensive care treatment<br>☐ other:<br>☐ unknown
38.	Secondary transport necessary	☐ no<br>☐ yes ⇨	name of secondary hospital
**VI**	**Patient's outcome**		
39.	Survival at last hospital discharge	☐ yes<br>☐ no ⇨	Overall ICU stay ___ days<br>Overall hospital stay ___ days
40.	Autopsy (non-survivors)	☐ yes ⇨<br>☐ no	Cause of death
41.	Findings at hospital discharge	☐ restitutio ad integrum<br>☐ movement restrictions<br>☐ paresis or plegia<br>☐ pain<br>☐ residual traumatic brain injury	☐ residual spinal cord injury<br>☐ cerebral performance category (CPC):<br>☐ other:<br>☐ unknown<br>List of injuries and impairments:
**VII**	**CRF completion**		
	Completed	date / /	
	Name	Signature	

Version 1.0 Page 2

Figure S3. Persons involved in via ferrata emergencies in Austria per year, itemized by cause of accident.

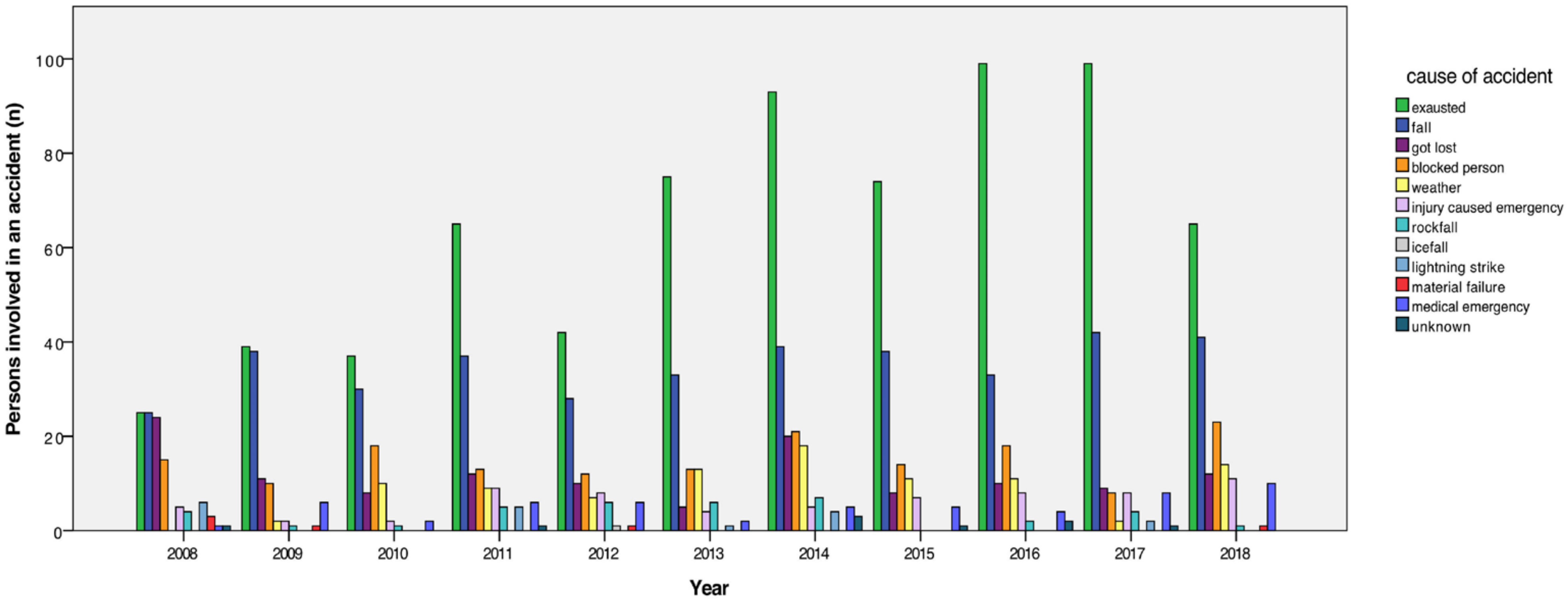

Table S1. Via ferrata grading using the Austrian Scale [4].

Via ferrata	Definition from the Austrian Scale
Grade A	Easy access. Many steps and footholds of good size available. No athletic passages. Itinerary fully equipped, many pegs available.
Grade B	Easy access. Athletic passages alternate with comfortable rests. The route is well equipped, but the spacing of steps or rungs requires good balance.
Grade C	Access is in mountain terrain and may require navigational skills. The itinerary is steep with many athletic passages with small holds. The equipment is safe, but spaced. It requires good physical and mental condition.
Grade D	The access, comparable with level C, may require short climbing sequences. The itinerary offers many exposed vertical or overhanging sections. The equipment is good, but with sections equipped only with cables.
Grade E	Access difficulties are the same as for Level D. The itinerary may have more physical and continuous sections than Level D. Equipment is limited to cables and anchors. No escape.

Table S2. Activity vs. injury classification in via ferrata emergencies from November 1, 2008 to October 31, 2018. Listed according to frequency.

Activity	Uninjured n (%)	Injured n (%)	Dead n (%)	Unknown n (%)	Total n (%)
Ascent	759 (57.6)	388 (29.5)	39 (3.0)	131 (9.9)	**1,317 (78.2)**
Descent	137 (56.4)	69 (28.4)	13 (5.3)	24 (9.9)	**243 (14.4)**
Traversing	25 (41.7)	20 (34.0)	6 (10.0)	9 (15.0)	**60 (3.6)**
Rappelling	4 (36.4)	4 (36.4)	1 (9.1)	2 (18.2)	**11 (0.7)**
Unknown	35 (66.0)	8 (15.0)	3 (5.7)	7 (13.3)	**53 (3.1)**
**Total**	**960 (57.0)**	**489 (29.0)**	**62 (3.7)**	**173 (10.3)**	**1,684 (100.0)**

Note: Unknown: police officers were unable to obtain information.

Table S3. Classification of via ferrata difficulty in relation to rescue efforts for via ferrata emergencies from November 1, 2008 to October 31, 2018.

Difficulty level of via ferrata	No rescue n (%)	Ground-based n (%)	Combined n (%)	Helicopter n (%)	Unknown n (%)	Total n (%)
Grade A	3 (3.9)	26 (34.2)	5 (6.6)	41 (53.9)	1 (1.3)	76 (4.5)
Grade B	14 (6.3)	88 (39.5)	20 (9.0)	99 (44.4)	2 (0.9)	223 (13.2)
Grade C	23 (6.0)	171 (44.4)	21 (5.5)	170 (44.4)	0	385 (22.9)
Grade D	19 (4.5)	188 (44.1)	48 (11.3)	167 (39.2)	4 (0.9)	426 (25.3)
Grade E	5 (4.2)	71 (60.2)	13 (11.0)	29 (24.6)	0	118 (7.0)
UIAA Grade	1 (0.8)	52 (39.7)	10 (7.6)	67 (51.1)	1 (0.8)	131 (7.8)
Undefined	18 (7.8)	97 (41.8)	7 (3.0)	108 (46.6)	2 (0.9)	232 (13.8)
Unknown	6 (6.5)	39 (41.9)	3 (3.2)	44 (47.3)	1 (1.1)	93 (5.5)
**Total**	**89 (5.3)**	**732 (43.5)**	**127 (7.5)**	**725 (43.1)**	**11 (0.7)**	**1,684 (100.0)**

Note: UIAA Grade was used when the terrain was defined according to alpine climbing UIAA Grading. Undefined designated the terrain whose difficulty was not defined by police or rescue teams. Unknown designated the situation when police officers were unable to obtain information.
